# Deferoxamine accelerates endothelial progenitor cell senescence and compromises angiogenesis

**DOI:** 10.18632/aging.203469

**Published:** 2021-09-11

**Authors:** Yi-Nan Lee, Hsueh-Hsiao Wang, Cheng-Huang Su, Hsin-I Lee, Yen-Hung Chou, Chin-Ling Hsieh, Wen-Ting Liu, Kuo-Tung Shu, Kai-Ting Chang, Hung-I Yeh, Yih-Jer Wu

**Affiliations:** 1 Cardiovascular Center, Department of Internal Medicine, MacKay Memorial Hospital, Taipei 10449, Taiwan; 2 Department of Medical Research, MacKay Memorial Hospital, Taipei 10449, Taiwan; 3 Department of Medicine, MacKay Medical College, New Taipei 25245, Taiwan; 4 Institute of Biomedical Sciences, MacKay Medical College, New Taipei 25245, Taiwan

**Keywords:** senescence, deferoxamine, angiogenesis, endothelial progenitor cell

## Abstract

Senescence reduces the circulating number and angiogenic activity of endothelial progenitor cells (EPCs), and is associated with aging-related vascular diseases. However, it is very time-consuming to obtain aged cells (~1 month of repeated replication) or animals (~2 years) for senescence studies. Here, we established an accelerated senescence model by treating EPCs with deferoxamine (DFO), an FDA-approved iron chelator. Four days of low-dose (3 μM) DFO induced senescent phenotypes in EPCs, including a senescent pattern of protein expression, impaired mitochondrial bioenergetics, altered mitochondrial protein levels and compromised angiogenic activity. DFO-treated early EPCs from young and old donors (< 35 vs. > 70 years old) displayed similar senescent phenotypes, including elevated senescence-associated β-galactosidase activity and reduced relative telomere lengths, colony-forming units and adenosine triphosphate levels. To validate this accelerated senescence model *in vivo*, we intraperitoneally injected Sprague-Dawley rats with DFO for 4 weeks. Early EPCs from DFO-treated rats displayed profoundly senescent phenotypes compared to those from control rats. Additionally, in hind-limb ischemic mice, DFO pretreatment compromised EPC angiogenesis by reducing both blood perfusion and capillary density. DFO thus accelerates EPC senescence and appears to hasten model development for cellular senescence studies.

## INTRODUCTION

The therapeutic potential of endothelial progenitor cells (EPCs) for ischemic diseases has attracted great interest since Asahara et al*.* first identified these cells in adult peripheral blood samples in 1997 [1]. The number of circulating EPCs is reduced in diabetes mellitus patients, is negatively associated with the Framingham cardiovascular risk score [2], and is positively associated with vascular function [1–3]. EPCs are needed not only for vascular repair, but also for the regulation of angiogenesis, and thus protect organs and tissues from critical ischemia in terminal atherosclerotic diseases [2, 4]. Clinically, EPCs have been widely used to treat patients with ischemic cardiovascular disease and diabetic foot ischemia [5].

Several studies have suggested that the EPC number decreases with age in humans and animals [6–8], possibly due to EPC senescence. Aging is an independent risk factor for the development of atherosclerosis-related diseases, so EPC senescence may accordingly promote atherosclerosis in elderly patients. As progenitor cells are more senescence-resistant than proper somatic cells, time-consuming procedures are needed to obtain sufficient quantities of senescent EPCs for routine assays. About one month of repeated replication is needed to obtain senescent EPCs *in vitro*, and about two years are needed to obtain them from old rats or mice *in vivo*.

Iron participates in a wide range of biological reactions, including enzyme activation, electron transfer, redox regulation, DNA damage repair and protein stabilization; thus, iron impacts cellular growth, energy metabolism and reactive oxygen species generation. Iron is required for the assembly, stability and function of respiratory complexes I, II and III for mitochondrial respiration, and also is important for the Krebs cycle, DNA metabolism and apoptosis [9]. Loss of iron homeostasis has been associated with accumulating oxidative damage and aging [10, 11], and increased reactive oxygen species levels due to iron deficiency can induce anemia, osteoporosis and cardiac disease [10–12].

Here, we pharmaceutically altered iron homeostasis using the Food and Drug Administration (FDA)-approved iron chelator DFO, in order to establish an accelerated EPC senescence model. We treated EPCs for a short period (four days) with a low dose (3 μM) of DFO, and determined its effects on EPC cell cycle arrest, mitochondrial bioenergetics, senescence-related protein expression, lipofuscin levels [13] and senescence-associated β-galactosidase (SA-βGal) activity. Then, we phenotypically compared these cells with EPCs that were isolated from old donors or repeatedly replicated. Our accelerated EPC senescence model may be suitable for both biochemical analyses and senolytic compound screenings.

## RESULTS

### Short-duration, low-dose DFO treatment is sufficient to induce EPC senescence and cell cycle arrest

**Figure 1:**
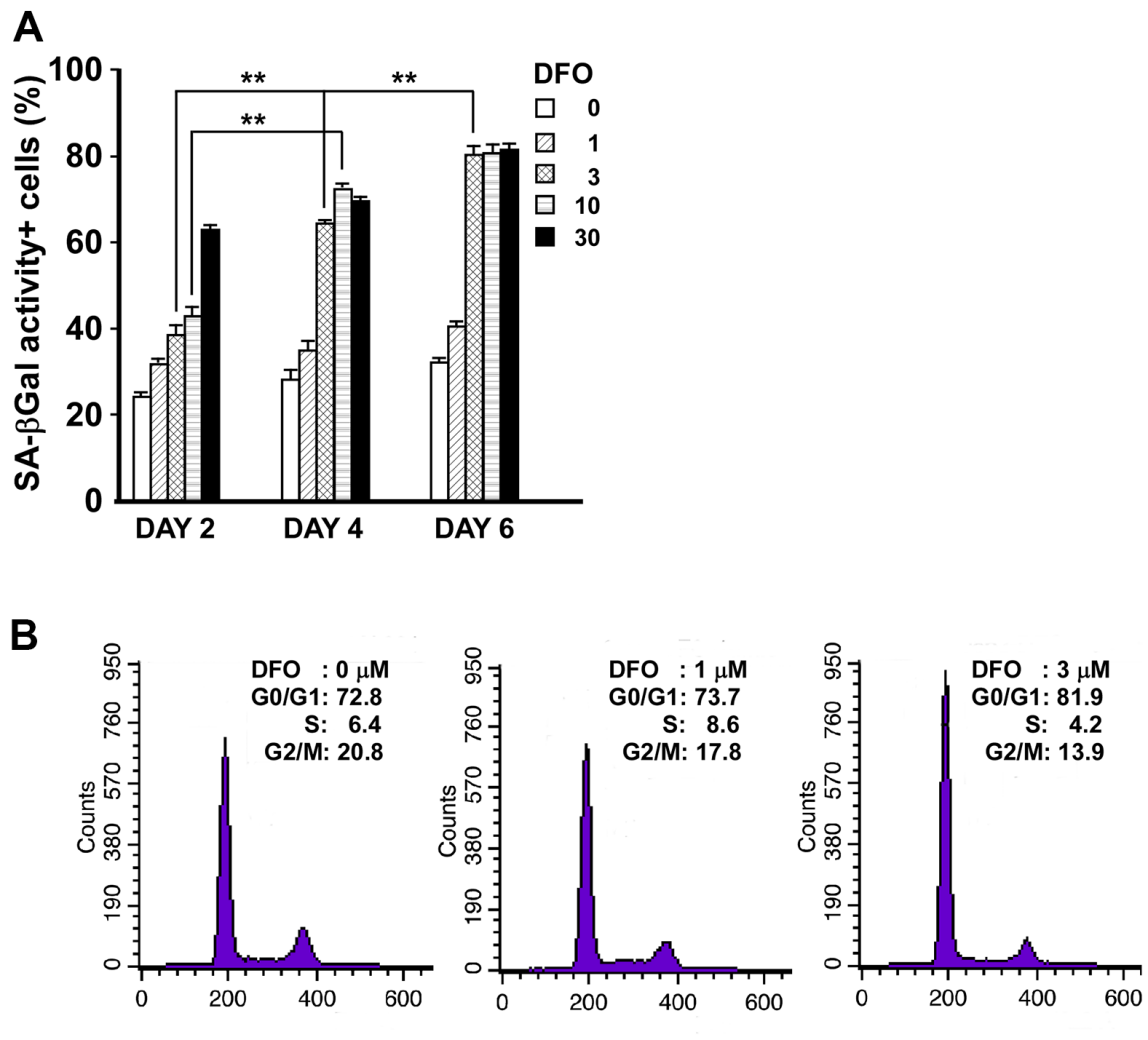
**Short-duration, low-dose DFO treatment is sufficient to induce EPC senescence and cell cycle arrest.** (**A**) DFO increased the percentage of SA-βGal activity-positive cells in a dose- and time-dependent manner. Late EPCs were treated with the indicated concentration (μM) of DFO for two, four or six days. Cells were fixed, stained with X-gal and quantified. ** P < 0.01 compared with the untreated group. N=3. (**B**) DFO increased the proportion of cells remaining in the G0 and G1 phases. The representative cell cycle analysis demonstrates that when different concentrations (0, 1 and 3 μM) of DFO were applied to the same clone of EPCs, there were graded increases in the percentages of cells from the same passage in G0 and G1 arrest, along with reduced percentages of cells in G2 and M phase. The experiment was repeated with three different clones of EPCs with similar results.

We first determined the duration and dose of DFO treatment needed to accelerate EPC senescence. Late EPCs were incubated with DFO at varying concentrations (0, 1, 3, 10 or 30 μM) for different incubation times (two, four or six days). Cell-based, quantitative measurements of senescence markers indicated that the percentage of SA-βGal activity-positive cells increased in a dose- and time-dependent manner upon DFO treatment (Figure 1A), and that 3 μM DFO treatment was sufficient to induce remarkable EPC senescence during a four-day culture process. Consistently, DFO-treated EPCs exhibited increased levels of lipofuscin, another well-accepted senescence marker [13] (Supplementary Figure 1). Likewise, at a low concentration (3 μM), DFO induced remarkable G1 arrest (Figure 1B), similar to that induced via serial passaging of EPCs (P8/P13, G0-G1: 67.0%/85.9%; G2-M: 26.9%/11.5%). Therefore, a low dose (3 μM) of DFO was sufficient to induce EPC senescence and cell cycle G1 arrest.

### DFO impairs EPC angiogenic activity

**Figure 2:**
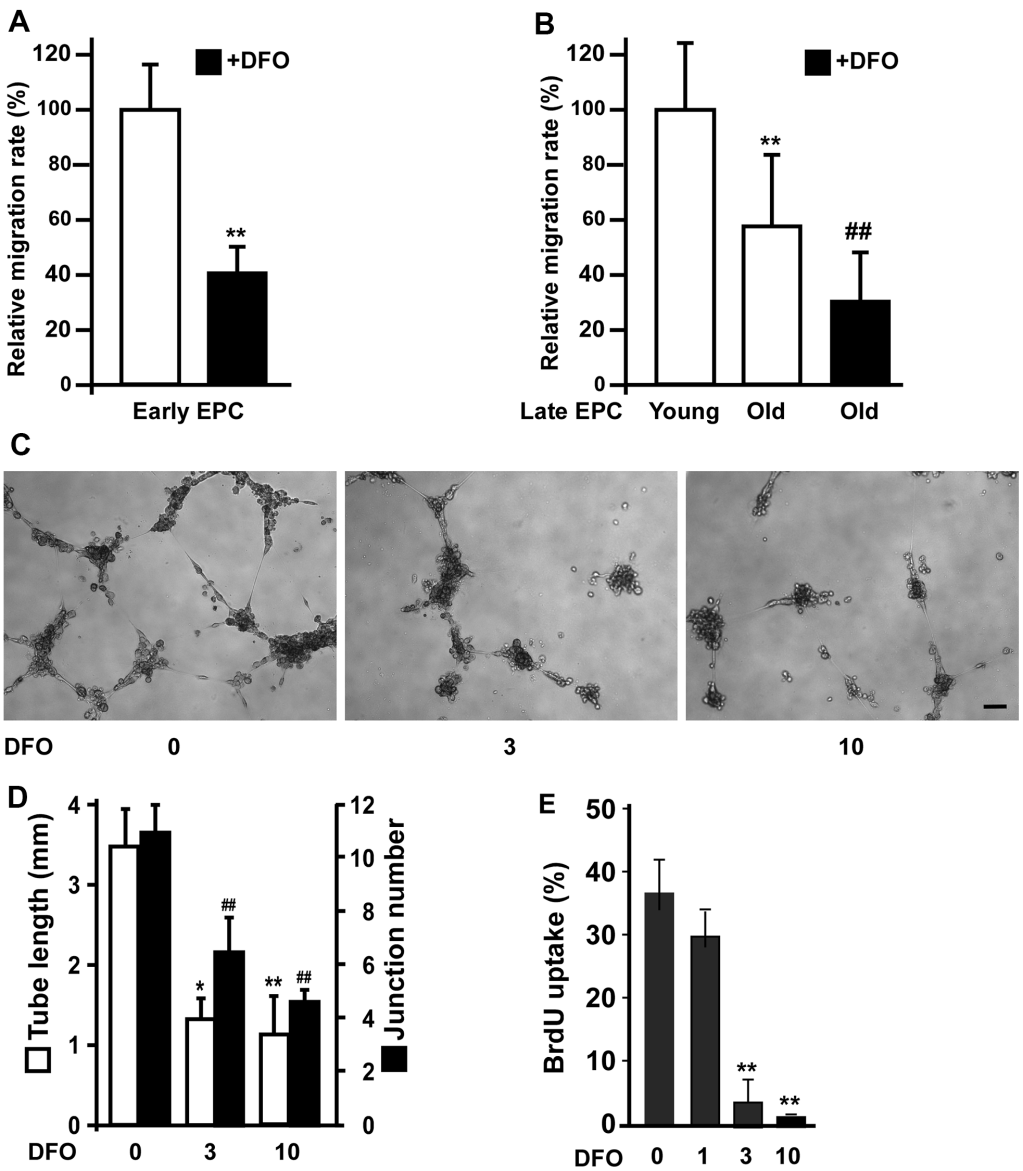
**DFO impairs EPC angiogenic activity.** (**A**) Early EPCs isolated from rat peripheral blood samples were treated with DFO (DFO, 3 μM) for four days, and then were subjected to migration assays. ** P < 0.01 compared with the untreated group. (**B**) Migration activity was reduced in old EPCs and further attenuated by DFO treatment. Human EPCs of the same clone with 10 additional passages were defined as old EPCs, as previously described [15]. Cells were treated with or without DFO (3 μM) for four days, and then were subjected to migration assays. ** P < 0.01 compared with the untreated group. (**C**) Late EPCs (1 × 10^4^) were incubated with the indicated concentration of DFO for four days, and were harvested for tube formation assays by being seeded in matrix gel overnight. (**D**) Quantification of the tube length and junction number. The experiment was repeated with five different clones of EPCs with similar results. * P < 0.05, ** P < 0.01 compared with the untreated group (0 μM). (**E**) EPCs were treated with the indicated concentration of DFO for four days, and then were subjected to a BrdU incorporation assay. ** P < 0.01 compared with the untreated group (0 μM).

We previously demonstrated that angiogenic activity was impaired in senescent EPCs [14]. Therefore, we examined the effects of DFO on EPC migration. DFO markedly reduced the migration of both early EPCs (Figure 2A) and late EPCs. Compared with young EPCs, old EPCs of the same clone exhibited impaired migration, and this was exacerbated by DFO treatment (Figure 2B). In addition, four-day DFO treatment dose-dependently inhibited EPC tube formation (assessed based on tube length and junction number; Figure 2C, 2D) and proliferation (Figure 2E).

### DFO induces a senescent protein expression pattern in EPCs

**Figure 3:**
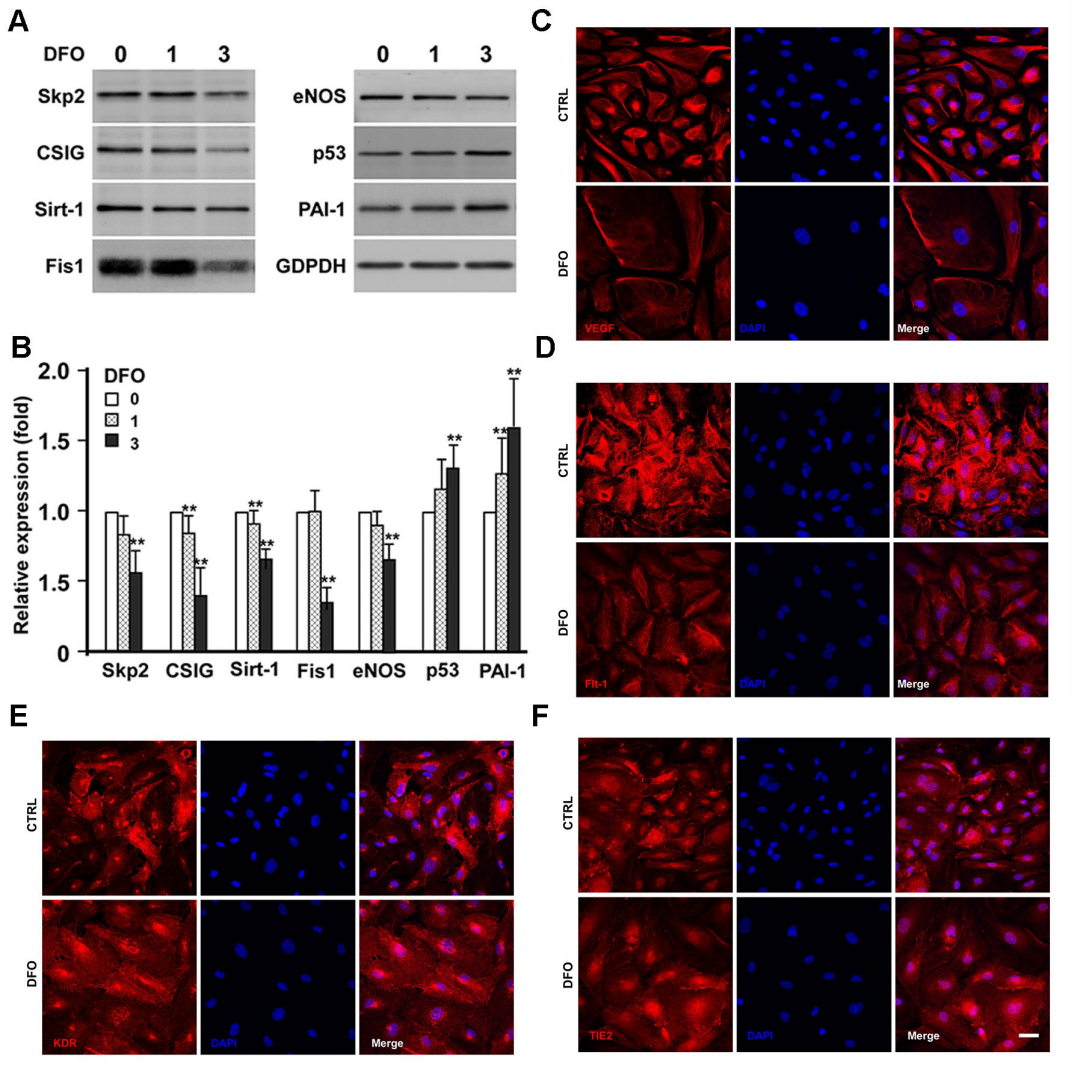
**Effects of DFO on the expression of senescence-related proteins and angiogenic factors.** (**A**) EPCs were treated with the indicated concentration (μM) of DFO for four days. Whole-cell lysates were harvested for electrophoresis, and Western blotting was performed with the indicated antibodies. (**B**) Quantification of relative senescence-related protein levels, normalized to GAPDH. Values are the mean ± standard deviation of triplicate assays from three independent experiments. ** P < 0.01 compared with the untreated group (0 μM). (**C**–**F**) Images are cells treated without (CTRL) and with DFO on the expression of VEGF, Flt-1, KDR and TIE2, respectively. Young EPCs were treated with 3 μM DFO for four days, and then were harvested for staining with the indicated antibodies. Scale bar, 50 μm.

As DFO increased the percentage of SA- βGal activity-positive cells and induced senescence-like G1 arrest, we next examined whether DFO induced a senescent protein expression pattern in EPCs. Four-day DFO treatment (at 0, 1 and 3 μM) dose-dependently reduced the expression of anti-senescence-related proteins such as S phase kinase-associated protein-2 (Skp2), cell senescence-inhibited gene (CSIG), sirtuin 1 (Sirt1) and mitochondrial fission 1 protein (Fis-1) (Figure 3A, 3B). More importantly, plasminogen activator inhibitor-1 (PAI-1) and p53 were significantly upregulated, while endothelial nitric oxide synthase (eNOS) was downregulated (Figure 3A, 3B). The DFO-altered protein expression patterns in the current study are highly mimicking the phenotypes induced by repeat replication as described [14–16].

Considering that DFO compromised the angiogenic activity of EPCs, we then examined whether four-day DFO treatment altered the expression of angiogenic proteins such as vascular endothelial growth factor (VEGF), Fms-related receptor tyrosine kinase 1 (Flt-1), kinase insert domain receptor (KDR) and TEK receptor tyrosine kinase (TIE2) in EPCs (Figure 3C–3F). DFO strongly downregulated VEGF, the major angiogenic factor in EPCs, consistent with the notion that EPCs primarily promote angiogenesis through their paracrine functions [17, 18]. Flt-1, KDR and TIE2 levels were also moderately reduced in DFO-induced senescent EPCs.

### DFO alters EPC mitochondrial bioenergetics and dynamics

**Figure 4:**
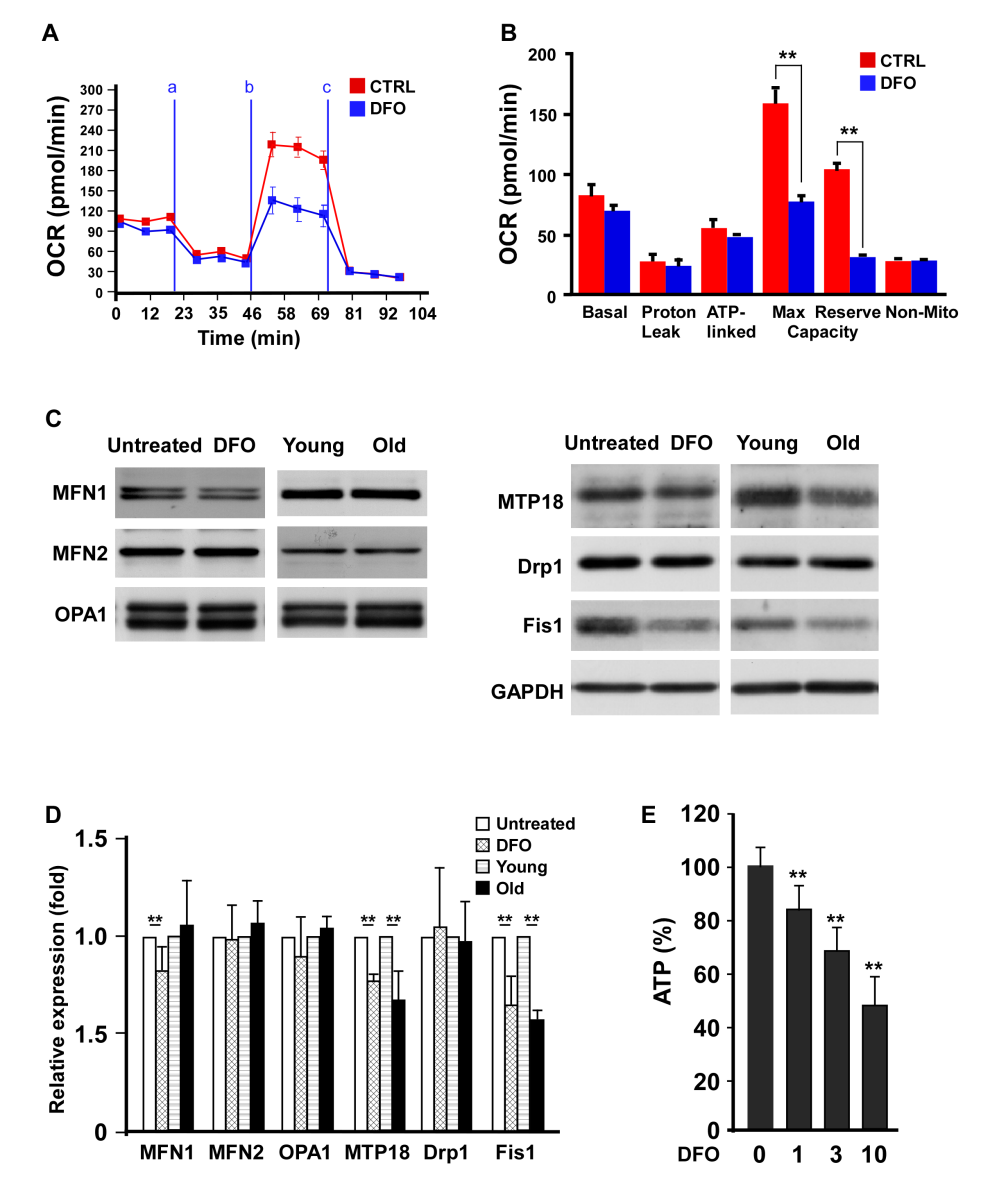
**Deferoxamine alters EPC mitochondrial bioenergetics and dynamics.** (**A**) DFO altered the bioenergetic profiles of human EPCs. Representative OCR curves of the EPCs are shown after the sequential addition of mitochondrial inhibitors to acquire respiratory parameters. The OCRs were automatically calculated and recorded in real time using Seahorse XF-24 software. EPCs with or without four-day 3 μM DFO treatment were seeded in a non-CO_2_ incubator for one hour before the OCR assay. Respiratory inhibitors were injected at the indicated times (a, oligomycin; b, CCCP; c, antimycin A) to determine the proton leak respiration, maximal respiratory capacity and mitochondrial reserve capacity, respectively. (**B**) Quantitative comparison of the OCRs of EPCs in the control (CTRL, 0 μM) and DFO (3 μM) groups. Despite proton leak respiration and non-mitochondrial respiration, all the OCR parameters were significantly reduced in the DFO group. * P < 0.05, ** P < 0.01. (**C**) Western blots were mitochondrial proteins in EPCs with or without DFO treatment, and in young or old EPCs. (**D**) Quantification of Western blot images are from three independent experiments. Young EPCs were treated with DFO (3 μM) for four days. EPCs of the same clone as the young EPCs with 10 additional passages were defined as old EPCs. (**E**) ATP production rate in DFO-treated EPCs. ** P < 0.01 compared with the untreated group. The experiment was repeated with three different clones of EPCs with similar results.

A previous report indicated that DFO treatment progressively reduced mitochondrial complex II and IV activity [19]. Accordingly, we examined the effects of DFO on mitochondrial respiratory efficiency in EPCs. The oxygen consumption rate (OCR) curve moved markedly downward in the DFO-treated group (Figure 4A), in a pattern highly resembling that of EPCs isolated from old animals [14]. Among the quantified respiratory parameters, the mitochondrial reserve capacity and the maximal respiratory capacity of the EPCs were significantly reduced upon DFO treatment (Figure 4B), indicating that DFO reduced the respiratory efficiency. Consistent with this finding, DFO diminished the adenosine triphosphate (ATP) production rate in EPCs (Figure 4E).

We also investigated the effects of DFO on proteins involved in mitochondrial dynamics. Two mitochondrial fission proteins, Fis1 and mitochondrial protein 18 kDa (MTP18), were significantly downregulated in old EPCs (replication-induced) compared with young EPCs, while fusion proteins such as mitofusin-1 (MFN1), MFN2 and optic atrophy 1 (OPA1) were not altered. Interestingly, chemical (DFO)- and replication-induced EPC senescence reduced Fis1 and MTP18 expression in the same pattern (Figure 4C, 4D).

### DFO-treated early EPCs from young and old donors exhibit similar senescent phenotypes

**Figure 5:**
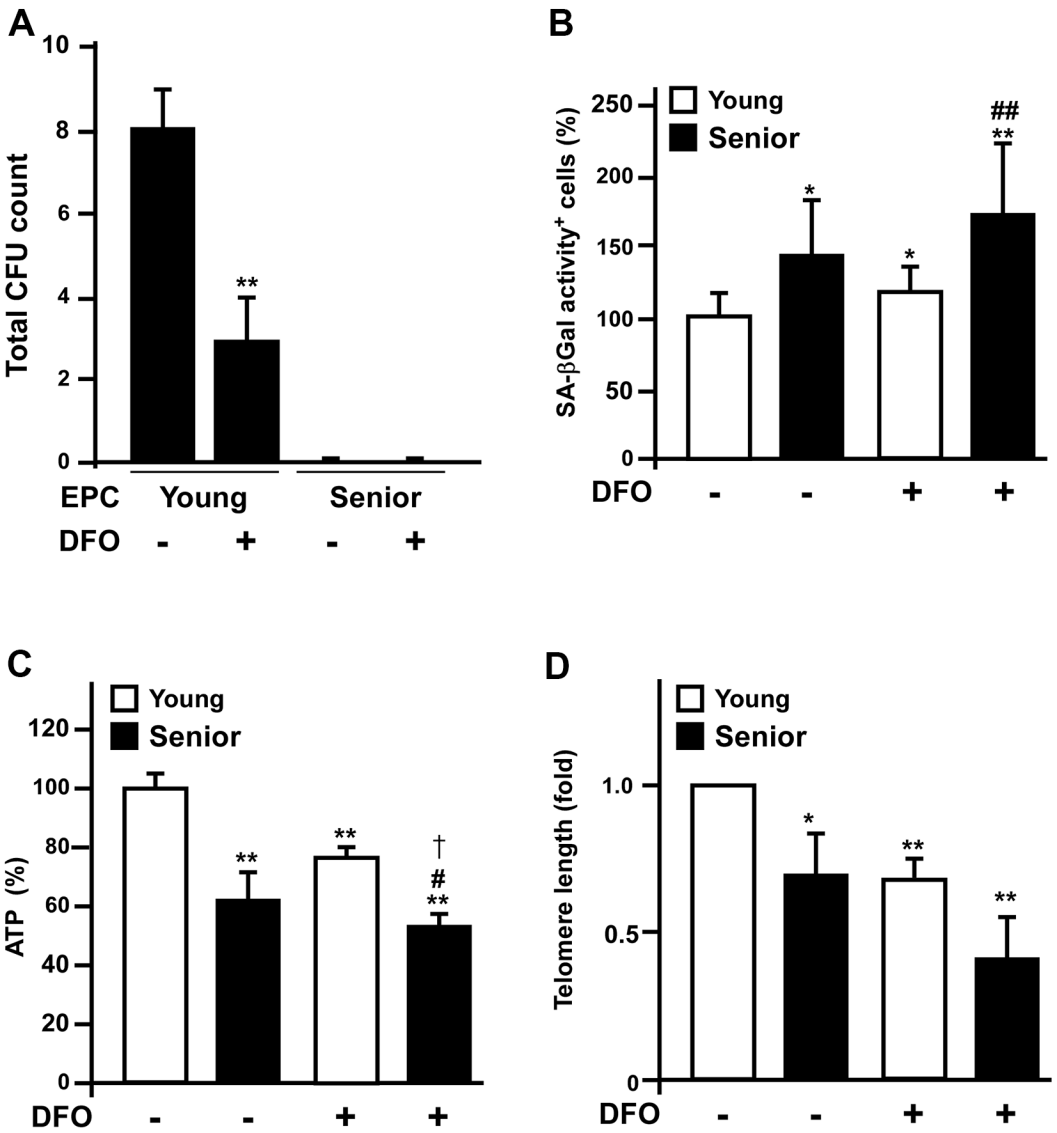
**Deferoxamine-treated early EPCs from young donors mimic the senescent phenotypes of those from old donors.** (**A**) Early EPCs were isolated from young (< 35 year-old) and senior (> 70 year-old) donors for colony formation assays. Early EPCs were incubated with or without deferoxamine DFO (3 μM) in a seven-day culture process. (**B**–**D**) Young and old EPCs of the same clone were treated with DFO (3 μM) for four days and then assessed for SA-βGal activity, ATP production and telomere length. * P < 0.05, ** P < 0.01 compared with the untreated young group; # P < 0.05, ## P < 0.01 compared with the untreated old group.

To evaluate whether *ex vivo* DFO treatment could mimic *in vivo* senescence, we harvested early EPCs from young (< 35 year-old) and senior (> 70 year-old) donors, and treated them with or without 3 μM DFO. We then assessed the colony-forming potential of the early EPCs, which is critical for their angiogenic activity. After seven days of culture, DFO dramatically reduced the colony-forming potential of early EPCs isolated from young donors; however, its effects on senior donors’ EPCs were blunted, as the colony-forming unit (CFU) counts of these cells were already low before DFO treatment (Figure 5A). However, compared to untreated groups, DFO is able to accelerate EPCs to develop other senescent phenotypes such as increased SA-βGal activity, reduced ATP production and diminished telomere lengths both in young and senior groups (Figure 5B–5D).

### DFO accelerates rat EPC senescence *in vivo*

**Figure 6:**
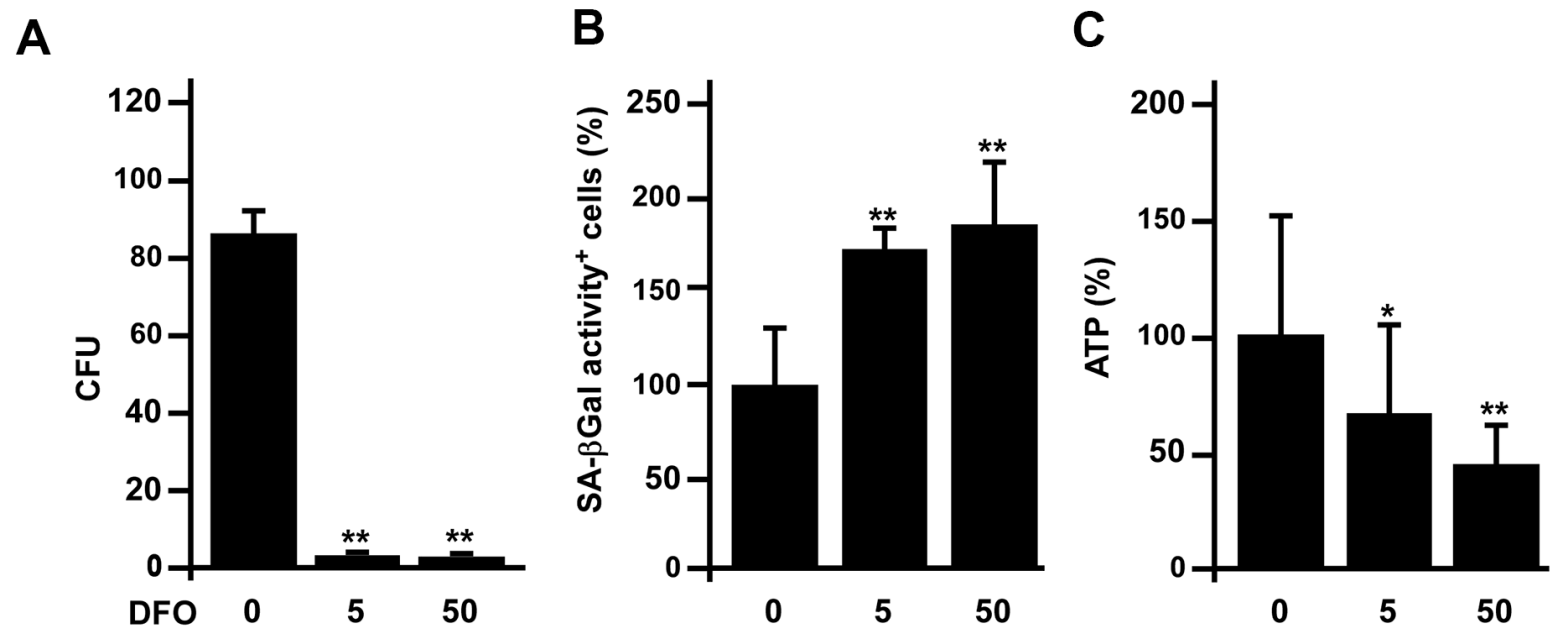
**DFO accelerates rat early EPC senescence *in vivo*.** (**A**–**C**) Three-month-old Sprague-Dawley rats were intraperitoneally injected with the indicated dose (mg/kg/day) of DFO for four weeks. Early EPCs were harvested for assessments of colony formation, SA-βGal activity and ATP production. * P < 0.05, ** P < 0.01 compared with the untreated group.

We also examined whether DFO could accelerate EPC senescence *in vivo*. Three-month-old Sprague-Dawley rats were administered DFO (0, 5 or 50 mg/kg/day) intraperitoneally for four weeks, and early EPCs were isolated from the rats for various assays. The results indicated that DFO pronouncedly reduced the colony formation (CFU count), increased the SA-βGal activity and reduced the ATP production of these cells (Figure 6A–6C).

### FeCl_3_ but not antioxidant rescues the DFO-induced senescent phenotypes and proliferation inhibition

**Figure 7:**
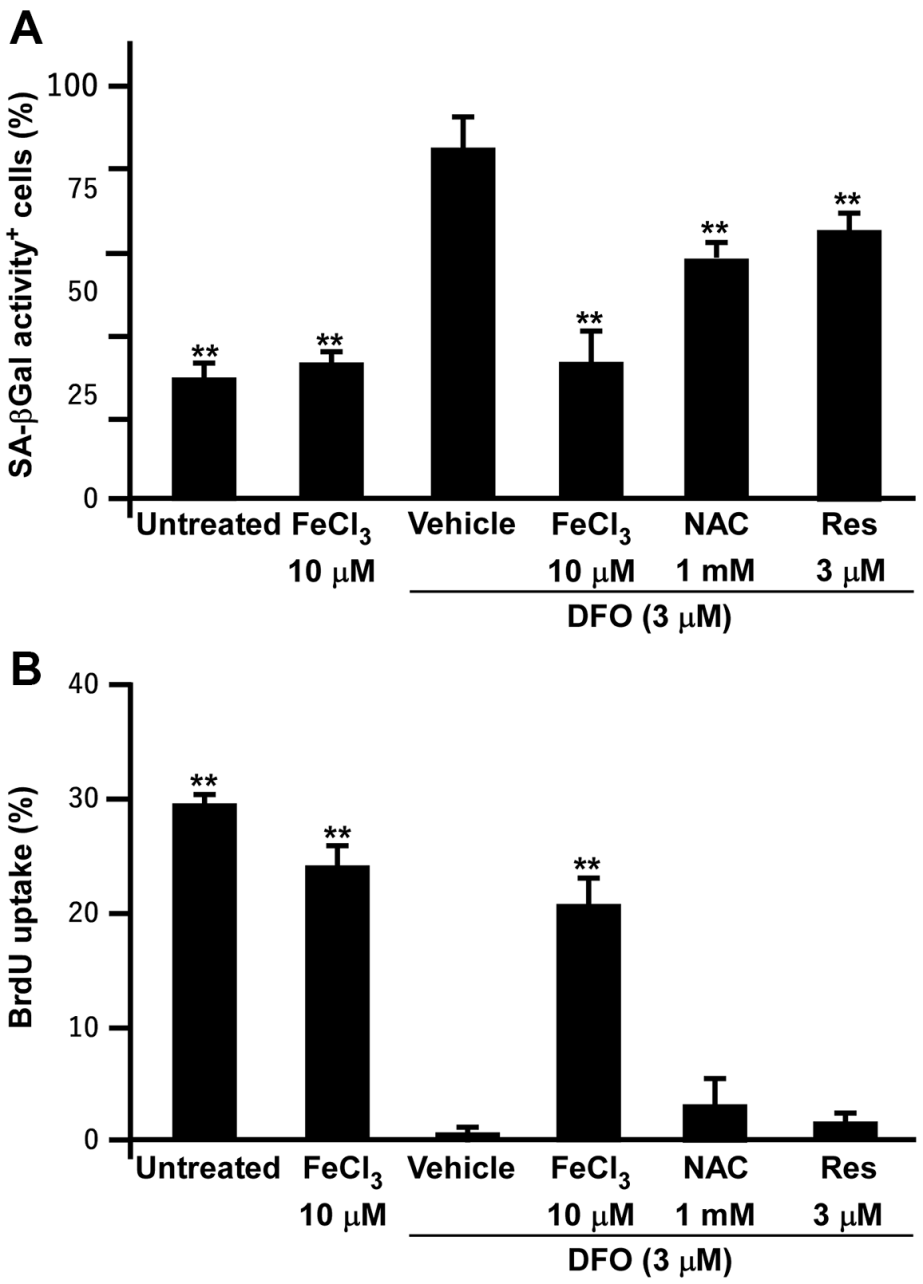
**FeCl_3_ but not antioxidant rescues the DFO-induced senescent phenotypes and proliferation inhibition.** (**A**) SA-βGal activity assay of EPCs. EPCs were treated with FeCl_3_ or DFO (3 μM) plus FeCl_3_, N-acetylcysteine (NAC), and Resveratrol (Res) as indicated concentrations for 4 days. (**B**) Iron but not antioxidants rescued DFO-induced proliferation inhibition. EPCs were treated with FeCl_3_ or DFO plus FeCl_3_ and antioxidants as described in (**A**). ** Compared with vehicle, P < 0.01.

As DFO induced senescence in EPCs and various cell lines, we tested whether the antioxidants N-acetylcysteine and resveratrol could prevent DFO-induced EPC senescence, using FeCl_3_ as a positive control. As expected, FeCl_3_ almost completely reversed the induction of senescence and the inhibition of proliferation in DFO-treated EPCs (Figure 7), indicating that this senescence model is iron-sensitive. However, both antioxidants only partially rescued the DFO-induced senescent phenotypes and failed to restore cell proliferation.

### The angiogenic activity of DFO-pretreated EPCs is compromised in hind-limb ischemic animals

**Figure 8:**
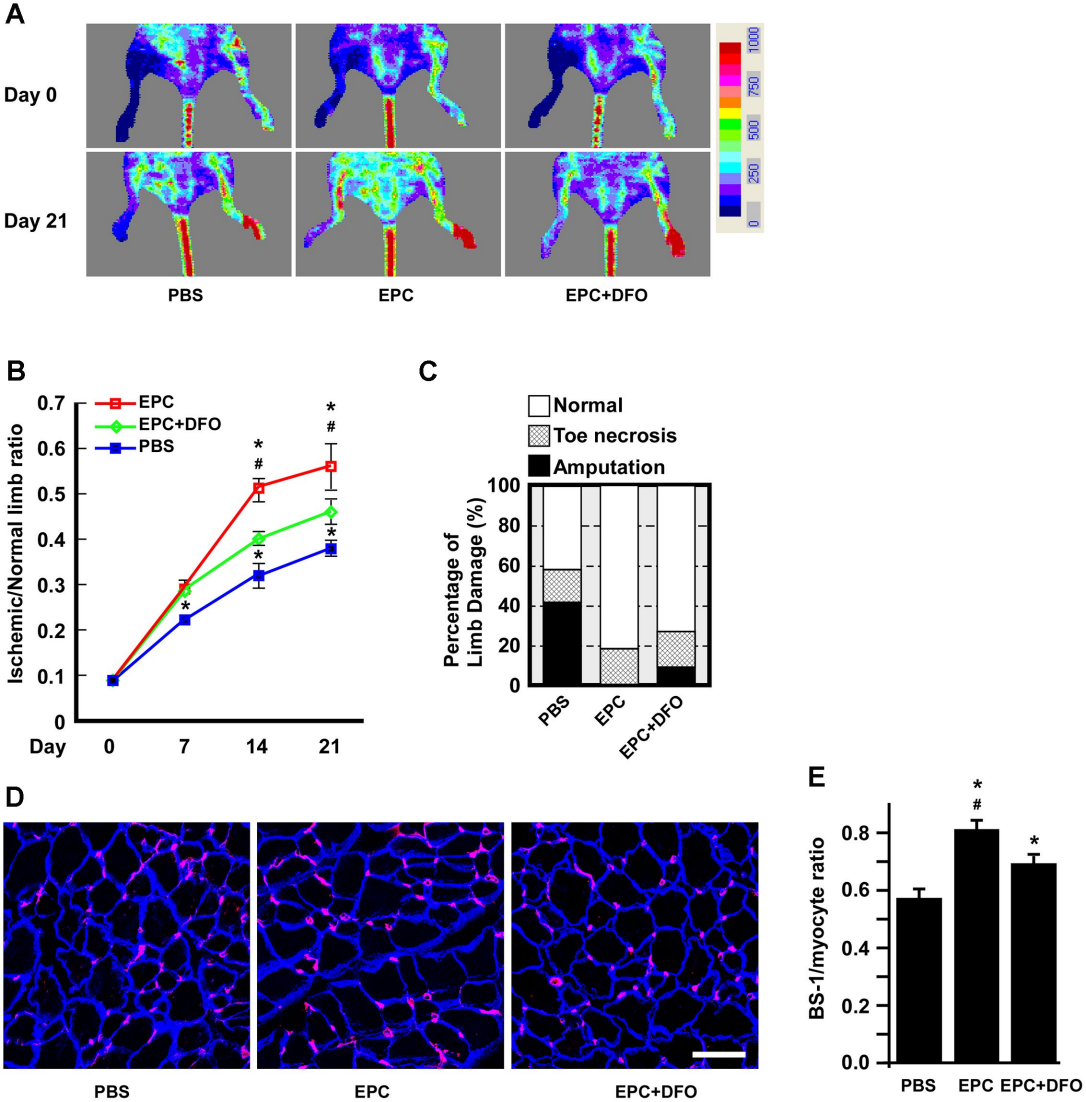
**The angiogenic activity of DFO-pretreated EPCs is compromised in hind-limb ischemic animals.** (**A**) Laser Doppler perfusion images of hind-limb ischemic rats injected with human EPCs to test their angiogenic activity. The rats were imaged on day 0 and day 21 after being injected with PBS, EPCs or EPCs treated with 10 μM DFO for four days. PBS without EPCs served as a mock injection, while untreated right hind limbs served as the control. (**B**) Quantification of the ischemic area versus the normal perfusion area. The days on which the images were captured are indicated. * P < 0.05 compared with the PBS group, # P < 0.05 compared with the DFO-treated EPC group. (**C**) Damaged tissues were rescued through the injection of young EPCs but not DFO-treated EPCs. The degree of tissue damage was classified into three categories: normal (n=10, empty box), toe necrosis (n=11, grid box) or amputation (n=11, filled box). The percentage of amputated limbs was 10% higher in the DFO-treated EPC group (EPC+DFO) than in the EPC-injected group. (**D**) Representative images of staining for myocytes and capillaries. On day 21, tissues were sectioned and stained with laminin (blue) and *Bandeiraea simplicifolia* lectin 1 (BS-I, red) to visualize myocytes and capillaries, respectively. Due to tissue atrophy, the peripheries of the PBS-treated myocytes appeared to be smaller than those of the EPC-injected myocytes. (**E**) Quantification of capillary density, which was calculated as the number of capillaries divided by the number of myocytes. * P < 0.05 compared with the PBS group, # P < 0.05 compared with the DFO-EPC group. Scale bar, 50 μm.

As short-duration, low-dose DFO treatment was sufficient to induce EPC senescence, we further evaluated the angiogenic activity of DFO-treated EPCs *in vivo* using a hind-limb ischemia model. Mice were subjected to hind-limb ischemia 24 hours before being injected with phosphate-buffered saline (PBS), EPCs or DFO-pretreated EPCs. Then, the subcutaneous perfusion of the hind limbs was evaluated using a laser Doppler imager (Figure 8A). Blood flow was restored in the EPC-injected group, whereas blood perfusion was apparently attenuated in the EPC+DFO group (Figure 8B). Ischemic limb recovery was also better in the EPC group than in the EPC+DFO group (Figure 8C); in fact, the amputation rate was 10% higher in the EPC+DFO group than in the EPC group. Consistently, the capillary density was also higher in the EPC group than in the PBS and EPC+DFO groups (Figure 8D, 8E). These results suggested that DFO pretreatment attenuated the angiogenic activity of EPCs *in vivo* in terms of perfusion, tissue damage and capillary density.

## DISCUSSION

Our study demonstrated that short-term (four days *in vitro* or four weeks *in vivo*), low-dose (3 μM) DFO treatment successfully induced EPC senescence *in vitro*, *ex vivo* and *in vivo*, closely mimicking the senescent phenotypes induced by repeated replication or natural aging [14]. These senescent phenotypes included G1 arrest, reduced proliferation and migration, impaired neoangiogenic (tube and colony formation) potential, enhanced senescent protein or biomarker expression, and inefficient mitochondrial dynamics and bioenergetics [14, 20]. To our knowledge, this is the first study to profile the effects of DFO on early and late EPCs. This accelerated EPC senescence model may substantially shorten the time required for many EPC senescence-related studies, particularly for the rapid screening of potential EPC senolytic agents.

It is well known that iron is essential for cell growth and survival, and iron overload is often observed in cancer cells. Many previous studies have demonstrated that iron deprivation can inhibit the proliferation of malignant cells; for example, in a hepatic cancer cell line, DFO was reported to promote senescence-like G1 arrest, reduce the cell growth rate and induce phenotypic biomarkers of replicative senescence (e.g., the typical flattened/enlarged morphology and enhanced SA-βGal activity) [21]. Here, we found that DFO induced late EPC growth arrest at G1 phase in a dose-dependent manner (Figure 1B), consistent with the results of previous studies in various cell lines [22–24]. In addition to altering the cell cycle transition, DFO induced EPC senescence in a dose- and time-dependent manner, as shown in quantitative assays of SA- βGal activity (Figure 1). Furthermore, DFO changed the expression of senescence-related proteins in EPCs (Figure 3 and Supplementary Figure 2) to a pattern perfectly identical to that of replication-induced senescent EPCs [14]. We previously found that these anti-senescence-related proteins, Skp2, CSIG, Sirt1 and Fis-1 were downregulated. Reduced Sirt1 expression, a more senescence-specific phenomenon, was detected both in our model and in miR-34a-induced EPC senescence [14, 25]. DFO induced senescence not only in late EPCs, but also in early EPCs obtained from rats and humans, as evidenced by increased SA-βGal activity and reduced CFUs, telomere lengths and ATP levels (Figures 5, 6).

In addition to elevated SA-βGal activity, increased lipofuscin expression is another well-accepted senescence marker [13]. Consistently, DFO increased lipofuscin levels in EPCs, suggesting that DFO accelerated senescence (Supplementary Figure 1). However, DFO was previously reported to prevent lipofuscin accumulation. Marzabadi et al*.* found that adding 30 μM ferric chloride (FeCl_3_) to the culture medium of rat cardiomyocytes increased lipofuscin levels, and this effect was enhanced when the ambient oxygen concentration was raised from 20% to 40%, but was counteracted when the cells were treated with high concentrations of DFO (ranging from 12.5 to 50 μM) long-term (up to 12 days) [26]. The conditions of our system differed from theirs in several ways. First, in our accelerated EPC senescence model, cells treated with low-dose DFO (3 μM) for four days exhibited senescent phenotypes (Figure 1) and increased lipofuscin levels (Supplementary Figure 1). Thus, FeCl_3_ supplementation and high oxygen concentrations were not needed to increase lipofuscin levels in our low-dose DFO-induced EPC senescence model. Second, EPCs are stem-like, actively proliferating cells, while cardiomyocytes are post-mitotic, differentiated cells. As iron is obligate for the proliferation of cell lines and hematopoietic progenitors [27], the impacts of iron depletion on stem-like EPCs and non-dividing cardiomyocytes are apparently different. Third, in breast cancer cell lines, even identical doses of DFO have been reported to have opposite effects on intracellular iron homeostasis [28, 29]. Lastly, as high concentrations of DFO can induce apoptosis [29], it may be that high-dose DFO treatment prevented lipofuscin accumulation by inducing cardiomyocyte apoptosis in the study of Marzabadi et al. Therefore, the effects of DFO on lipofuscin accumulation and intracellular iron levels may depend on the cell type (stem cell-like vs. post-mitotic) and treatment dosage.

The best-recognized hypothesis of aging was developed in the 1950s by Harman, who stated that aging is the accumulation of damage resulting from excessive oxidative stress [30]. The antioxidant effects of DFO have been reported for decades; however, these effects are unrelated to its iron-chelating properties [31, 32]. By using DFO-induced EPC senescent model to test the effects of antioxidants on cell senescent process, we find that only FeCl_3_ supplement rescues the DFO-induced senescent phenotypes and proliferation inhibition (Figure 7). Applying antioxidants only partially rescued the DFO-induced senescent phenotypes and failed to restore cell proliferation. Thus, the current model closely mimics the natural aging process, which is intricately regulated and cannot be reversed by free radical quenching alone. In agreement with these data, previous studies have indicated that antioxidative enzyme overexpression fails to extend the lifespans of *C. elegans*, fruit flies and mice [33–35].

Our model also simulated natural senescence in terms of mitochondrial dynamics and bioenergetics (Figure 4). DFO has been shown to reduce mitochondrial complex II and IV activity in other cell types [19, 36]. By monitoring the real-time OCR, we found that DFO induced a senescent pattern of mitochondrial bioenergetics in EPCs, identical to the pattern observed in EPCs isolated from old rats or subjected to replication-induced senescence [14]. Moreover, we previously demonstrated that EPC senescence could largely be attributed to the downregulation of the mitochondrial fission protein Fis1 and the accompanying changes in the mitochondrial network, whereas ectopic Fis1 expression significantly rejuvenated replication-induced senescent EPCs [20]. In the present study, the mitochondrial dynamic protein expression pattern of DFO-treated EPCs was highly similar to that of replication-induced senescent EPCs, characterized by minor changes in fusion proteins but significant decreases in the fission proteins Fis1 and MTP18 (Figure 4C, 4D).

Several studies have demonstrated that senescence impairs the angiogenic activity of EPCs, thus increasing the risk of cardiovascular disease in the elderly. Indeed, we previously found that replication-induced senescent EPCs exhibited impaired tube formation, as well as diminished neoangiogenesis and perfusion recovery in hind-limb ischemic nude mice [20]. In this study, we observed that DFO dose-dependently attenuated the tube formation capacity of EPCs (Figure 2C, 2D). To determine whether DFO could also accelerate early EPC senescence, we harvested early EPCs from young (< 35 year-old) and old (> 70 year-old) donors. The EPCs from old donors nearly lost their colony-forming potential, indicating a poor ability to enhance angiogenesis [37]. Short-term *in vitro* DFO treatment also significantly attenuated colony formation in early EPCs from young donors (Figure 5A). Likewise, injecting Sprague-Dawley rats with DFO impaired the colony-forming potential of early EPCs *in vivo* (Figure 7A). DFO enhanced early EPC senescence not only by reducing the CFU count, but also by increasing SA-βGal activity, reducing ATP production and shortening the telomere length both *in vitro* and *in vivo* (Figures 5B–5D, 6B, 6C). Interestingly, several studies have shown that locally injected DFO is more of an angiogenic agent than an anti-angiogenic agent [38–40], likely because it is a hypoxia-mimetic agent that activates hypoxia inducible factor 1 alpha-dependent pathways to induce neovascularization [41, 42]. Therefore, local DFO injection may promote neovascularization by indirectly recruiting mature endothelial cells *in situ,* even though DFO actually reduces the angiogenic potential of circulating EPCs per se. Our present results indicated that DFO pretreatment indeed attenuated the angiogenic activity of EPCs in a hind-limb ischemic model (Figure 7).

To the best of our knowledge, this study is the first to establish an accelerated EPC senescence model using DFO. DFO-treated EPCs displayed canonical senescent phenotypes, including increased SA-βGal marker levels, altered senescence-related protein levels and impaired angiogenic activity. Our results indicate that establishing accelerated EPC senescence models *in vitro* and *in vivo* may expedite the very time-consuming study of EPC senescence.

## MATERIALS AND METHODS

### Human late EPC isolation and characterization

This study was approved by the Mackay Memorial Hospital Institutional Review Board (ethical approval no. MMHIS566). All participants provided written informed consent, and all methods were performed in accordance with the ethically approved protocol. Human late EPCs were isolated according to a previously described method [14]. In brief, peripheral blood mononuclear cells were isolated from peripheral blood samples (80 mL) from healthy donors through centrifugation on Ficoll-Paque™ Plus (GE Healthcare, USA) in accordance with the manufacturer’s instructions. CD34^+^ cells were further isolated from the peripheral blood mononuclear cells using a CD34 MicroBead kit and MACSTM Cell Separation System (both from Miltenyi Biotec, Germany), and were maintained using an endothelial cell growth medium MV2 kit (PromoCell, Germany). Cells (1 × 10^6^ cells/cm^2^) were seeded on fibronectin-coated dishes (BD Biosciences, USA) supplemented with endothelial cell MV2 medium, and were incubated in a 5% CO_2_ incubator at 37° C.

The purified EPCs were characterized using qualified antibodies, as shown in Supplementary Figure 3. EPCs were further characterized based on their uptake of 1,1'-dioctadecyl-3,3,3',3'-tetramethyl-indocarbocyanine perchlorate acetylated-low density lipoprotein (DiI-acLDL), their binding to Ulex europaeus agglutinin-1 (UEA-1) lectin, and their expression of KDR, eNOS and VEGF. Old EPCs were obtained through the repeated replication of young EPCs (~passages 7-8) until their cell doubling times were twice as long as those of young EPCs from the same clone.

### Early EPC isolation and colony formation assay

Early EPCs were isolated as previously described [43], with some modifications. In brief, 80-mL peripheral blood samples were collected from healthy donors for the isolation of peripheral blood mononuclear cells. For rat EPCs, peripheral blood was obtained from the heart immediately before sacrifice. Ficoll-Paque™ Plus gradients were used during centrifugation to separate the fraction of mononuclear cells from other blood components, in accordance with the manufacturer’s instructions. Mononuclear cells in the low-density fraction were harvested and washed twice with PBS-ethylenediaminetetraacetic acid (2 mM). Purified mononuclear cells (1 × 10^6^ cells/cm^2^) were grown on fibronectin-coated dishes (BD Biosciences) supplemented with the EGM-2 Bullet Kit system (Lonza, Switzerland), which consists of endothelial basal medium, 2% fetal bovine serum, human epidermal growth factor, human fibroblast growth factor-B, insulin-like growth factor-1, ascorbic acid and heparin. The cells were incubated in a 5% CO_2_ incubator at 37° C. The medium was changed every three days, and each cluster or colony was visually inspected daily through an inverted microscope at 40X magnification.

### Animal experiments

All animal experiments and protocols were approved by the Institutional Animal Care and Use Committee of Mackay Memorial Hospital (approval numbers: MMH-A-S-970-13 and A1000003). Experiments were performed on 8- to 10-week-old male Sprague-Dawley rats (National Laboratory Animal Center, Taiwan) maintained under a 12-hour light/dark cycle. Standard laboratory chow and water were available ad libitum.

### SA-βGal activity measurement

SA-βGal activity was quantified using a senescence assay kit (Abcam, UK) in accordance with the manufacturer’s instructions. EPCs (5 × 10^3^ cells/well) were seeded on a 24-well plate in complete cell culture medium with or without 200 nM daunorubicin HCl at 37° C with 5% CO_2_. After 48 hours of incubation, the medium was replaced with senescence dye containing buffer, and the cells were incubated for two hours. The cells were then washed twice, trypsinized and analyzed using fluorescence-activated cell sorting.

Senescent cells were imaged using a β-galactosidase staining kit (BioVision, USA) according to the manufacturer’s instructions. EPCs (1 × 10^4^) in 24-well plates were washed with PBS and fixed with 0.5 mL of fixative solution for 15 min at room temperature. After being washed, the cells were incubated overnight with X-gal (1 mg/mL) at 37° C. The cells were then washed again and observed under a microscope at 200X magnification so that blue-colored positive cells could be counted.

### Measurement of cellular ATP production

Intracellular ATP concentrations were measured using an ATP Bioluminescence Assay kit CLS II (Roche, # 11 699 695 001) in accordance with the manufacturer’s instructions. Total cell lysates from 1 × 104 EPCs were resuspended in 100 μL of dilution buffer. An ATP standard curve in the range of 10–5 to 10–10 M was prepared using serial dilutions of an ATP standard solution in redistilled water. The total cell lysates were diluted in nine volumes of a boiling 100 mM Tris, 4 mM ethylenediaminetetraacetic acid solution (pH 7.75). The samples were incubated for 2 min at 100° C and then centrifuged at 1000 × g for 60 sec. The supernatants were transferred to fresh tubes, and luciferase reagent was added to the samples and standards. ATP-catalyzed bioluminescence was then measured on a luminometer and integrated for 1 to 10 sec. Intracellular ATP concentrations were calculated from a log-log plot of the standard curve data.

### Cell growth and proliferation assay

Cell proliferation was evaluated based on nuclear bromodeoxyuridine (BrdU) incorporation using a BrdU immunochemistry kit (Millipore, USA). EPCs (1.5 × 10^4^ cells/well) were grown on coverslips in a 24-well plate overnight. Six hours before the assay, the cells were incubated with 10 μM BrdU. The cells were then washed twice and fixed with ice-cold 70% ethanol at 4° C for 30 min. The cells were washed with PBS, blocked with 10% fetal bovine serum and incubated for two hours with an anti-BrdU antibody. After being washed three times, the cells were incubated with horseradish peroxidase-conjugated antibodies. Finally, the cells were visualized using 3,3’-diaminobenzidine tetrahydrochloride staining according to the manufacturer’s instructions.

### Cell cycle analysis

EPCs (1 × 10^6^) were harvested and resuspended in single-cell suspension buffer (PBS + 2% fetal bovine serum). After being washed, the cells were fixed with ice-cold 70% ethanol and then incubated with a propidium iodide solution (50 μg/mL propidium iodide, 0.1 mg/mL RNase A, 0.05% Triton X-100) at 37° C for 40 min. The cells were pelleted, resuspended in 500 μL of PBS and analyzed using flow cytometry on a FACScan flow cytometer (BD Biosciences).

### Mitochondrial functional evaluation based on the OCR

The cellular OCR (pmol/min) was evaluated as a measure of mitochondrial function on a Seahorse Bioscience XF24 extracellular flux analyzer (Bucher Biotec AG, Switzerland). On the day of metabolic flux analysis, cells were incubated at 37° C in a non-CO_2_ incubator for one hour with customer-formulation Endothelial Cell BM MV2 medium (c-97139, PromoCell). First, the baseline cellular OCR was measured. For mitochondrial respiration analysis, ATP synthase was inhibited using the mitochondrial inhibitor oligomycin (10 μM, Sigma, USA). For the analysis of the mitochondrial membrane potential and electron transport chain, the protonophore carbonyl cyanide m-chlorophenyl hydrazine (CCCP, 50 μM, Sigma) and antimycin A (5 μM, Sigma) were injected as inhibitors. The relative contributions of the basal, non-mitochondrial, ATP-linked, proton leak-linked and maximal OCRs were calculated, along with the cell reserve capacity, and progress curves were plotted.

### Relative telomere length determination

EPCs (1 × 10^5^) were harvested for genomic DNA extraction to determine telomere length. PCR was conducted with a mixture of 270 nM telomere sense primers, 900 nM telomere antisense primers and 1× SYBR Green Master Mix (Roche, USA). The primer sequences used for telomere length determination were: telomere sense strand, GGTTTTTGAGGGTGAGGGTGAGGGTGAGGGTGAGGGT; telomere antisense strand, TCCCGACTATCCCTATCCCTATCCCTATCCCTATCCCTA. The internal control sequences were: 36B4 antisense strand, CCCATTCTATCATCAACGGGTACAA; 36B4 sense strand, CAGCAAGTGGGAAGGTGTAATCC. The reaction proceeded for one cycle at 95° C for 5 min, followed by 25 cycles of 95° C for 15 sec, 54° C for 2 min, and a final extension at 72° C for 5 min. Quantitative PCR was performed on an ABI One Step Real-Time PCR machine (Applied Biosystems, USA). We calculated the relative telomere length (normalized T/S ratio) using the comparative Ct method after verifying that the quantitative PCR results for telomeres and 36B4 had equivalent amplification efficiencies.

### Western blotting analysis

Radioimmunoprecipitation assay buffer was used to harvest total cell lysates from EPCs. The cell lysates (50 μg/sample) were resolved using sodium dodecyl sulfate polyacrylamide gel electrophoresis, and then were transferred onto polyvinylidene fluoride membranes (Bio-Rad, USA) at 4° C overnight. The blots were blocked with 10% bovine serum albumin and then incubated with primary antibodies (diluted 1:1000) for two hours at room temperature to detect proteins associated with senescence, the cell cycle, mitochondrial function or endothelial cell function. The antibodies included Sirt1 (Sigma), CSIG (GeneTex, USA), Skp2 (Cell Signaling Technology, MA, USA), p53 (Epitomics, USA), MFN1 (Santa Cruz Biotechnology, USA), MFN2 (Cell Signaling Technology), OPA1 (Cell Signaling Technology), MTP18 (Abcam), Drp1 (Santa Cruz), Fis1 (Sigma), eNOS (BD Biosciences) and PAI-1 (Abcam). Corresponding secondary antibodies conjugated with alkaline phosphatase were used, and the chemiluminescence reaction was conducted using VisiGlo substrate (Amresco, USA) in accordance with the manufacturer’s instructions. The blots were scanned and densitometric analyses were performed using TotalLab software (Nonlinear Dynamics).

### Hind-limb ischemia in nude mice and EPC angiogenesis

To create ischemic hind limbs, we anesthetized female BALB/c athymic nude mice (8 weeks old, weighing 18-22 g) by intraperitoneally injecting them with pentobarbital (80 mg/kg). The right femoral artery and vein were ligated and severed from just above the deep femoral arteries to the popliteal artery and vein. Twenty-four hours after surgery, the right thighs and calves of the mice were injected with one of the following reagents: 1) 50 μL of PBS alone (PBS group), 2) PBS containing young EPCs (2 × 10^5^, EPC group), or 3) PBS containing young EPCs (2 × 10^5^) pretreated with 10 μM DFO for four days (EPC+DFO group). After 21 days, the mice were sacrificed with an overdose of intraperitoneal pentobarbital (100 mg/kg), and their calf muscles were dissected and processed for immunohistochemical analysis.

### Laser doppler perfusion imaging

The subcutaneous perfusion of the hind limbs was evaluated using a laser Doppler imager (Moor Instruments, UK). Mice without amputated hind limbs were anesthetized with pentobarbital (80 mg/kg, intraperitoneal) and placed on a heater at 37° C for 10 min before their ischemic legs and feet were scanned. Laser Doppler imaging was performed 24 hours after surgery (day 0, just before the injection of EPCs). The same procedures were performed on days 7, 14 and 21 after EPC injection. The subcutaneous perfusion status of the right (ischemic) hind limb was expressed relative to that of the left (normal) hind limb.

### Immunofluorescence

Confluent EPCs on cover glasses were fixed with 4% paraformaldehyde (electron-microscopy grade, Electron Microscopy Sciences, USA) for 10 min and then washed three times with PBS. The cells were blocked with 10% horse serum for one hour and incubated with primary antibodies (all diluted 1:100) at 4° C overnight. The following antibodies were used: platelet-endothelial cell adhesion molecule-1 (PECAM1, CD31; MA3105; Invitrogen), VE-cadherin (sc-9989; Santa Cruz), Flt-1 (ab2350; Abcam), TIE2 (ab24859; Abcam), eNOS (32027; Cell Signaling Technology), KDR (2479S; Cell Signaling Technology), VEGF (1909-S; Epitomics) and vWF (AHP062; Bio-Rad). After being washed three times with PBS-Triton X-100 (0.2%), the cells were incubated with the corresponding secondary antibodies at room temperature for three hours. Cell nuclei were stained with 4′,6-diamidino-2′-phenylindole (DAPI, 1 mg/mL, diluted 3000X) for 10 min and then washed three times with PBS-Triton X-100 (0.2%). The cells cultured on cover glasses were mounted with ProLong^™^ Mountant (Invitrogen, USA) and imaged with a Leica TCS SP8 confocal microscope (Germany).

Calf muscles were placed in 30% sucrose-PBS for 24 hours, bisected at the middle level, mounted in an optimal cutting temperature compound (Tissue-Tek) and snap-frozen in liquid nitrogen. For the capillary density analysis, the calf muscles were mid-bisected, with one cross-section 1.5 mm above the bisecting plane and another cross-section 1.5 mm below the bisecting plane, so that the two sections were 3 mm apart. Both sections were fixed with methanol for 10 min, washed briefly with PBS, blocked with 10% horse serum, and labeled with a mixture of a monoclonal rat anti-murine PECAM1 (CD31) antibody (1:200; B and D Pharmingen, USA) and a polyclonal rabbit anti-laminin antibody (1:100; Chemicon) at 37° C for two hours. The sections were then incubated with a mixture of a CY3-conjugated anti-rat antibody and a CY5-conjugated anti-rabbit antibody (both from Chemicon). In parallel, methanol-fixed frozen sections were stained with tetramethylrhodamine isothiocyanate-conjugated murine EC-specific *Bandeiraea simplicifolia* lectin 1 (BS-I, 1:50; Sigma) and a polyclonal rabbit anti-laminin antibody (1:100; Chemicon) at 4° C overnight, and then were incubated with a CY5-conjugated anti-rabbit antibody. Capillaries were counted in 30 randomly chosen high-power fields on the two sections from each animal (without amputated hind limbs). The results are expressed as capillaries per myocyte.

### Statistical analyses

Values are expressed as the mean ± standard deviation. Student’s *t* test was used to assess differences between the means of two groups, while analysis of variance was used to assess differences among the means of three or more groups. A linear regression or curve-fitting test was used to assess the correlation between two variables. P values < 0.05 were considered statistically significant.

## Supplementary Material

Supplementary Figures
