# Supplementary material for: Deferoxamine accelerates endothelial progenitor cell senescence and compromises angiogenesis
Source: Aging (Albany NY). 2021 Sep 11;13(17):21364–84. doi: 10.18632/aging.203469 (PMC8457614; doi:10.18632/aging.203469)
Supplement: Supplementary Figures [file aging-13-203469-s001.pdf]

SUPPLEMENTARY FIGURES

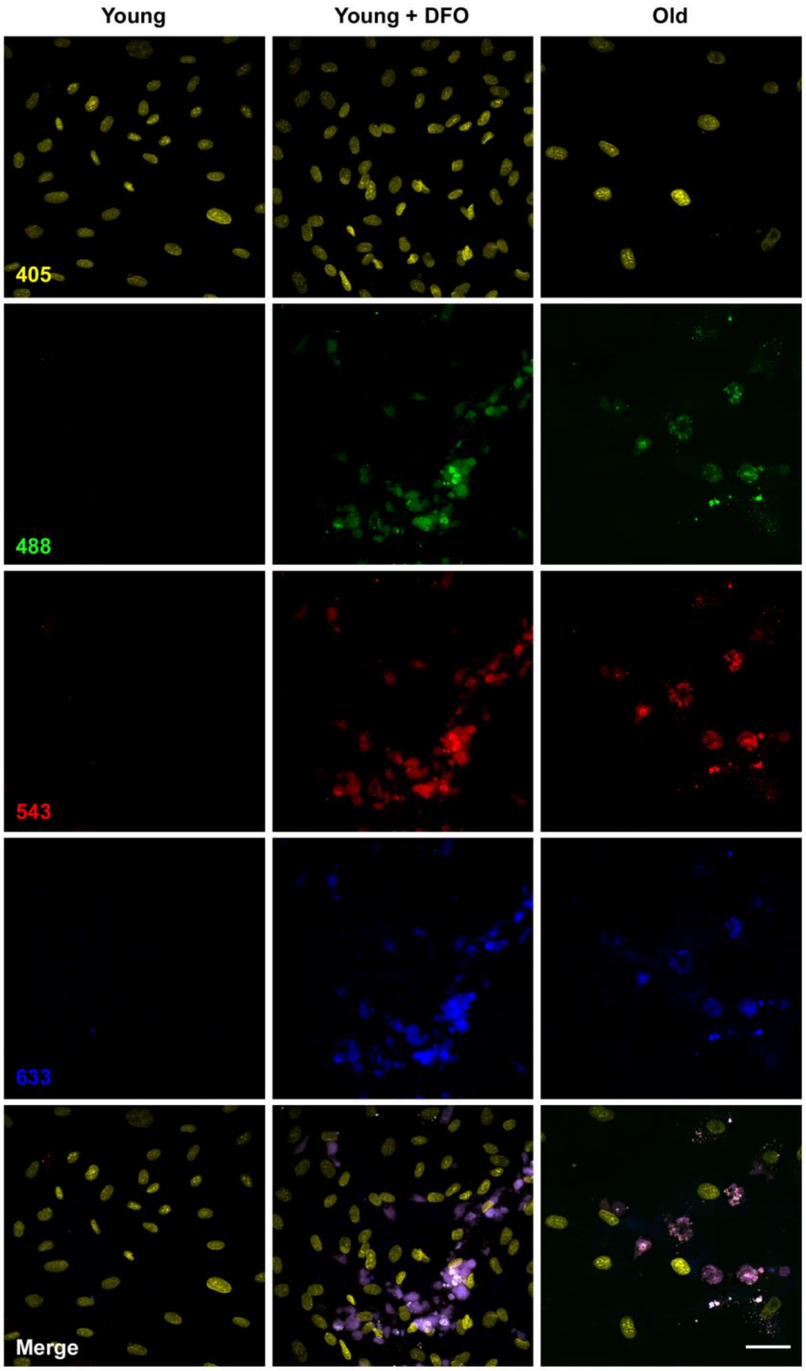

Supplementary Figure 1. DFO accelerates cell senescence with an increase of lipofuscin. Young EPCs (P9) were treated with DFO (3 µM) for 4 days. Cells were fixed with ice-cold methanol at -20° C for 5 min. After 3 times of PBS washes, nuclei were stained with DAPI for 10 min. Images were scanned with 405 nm (UV) to excite DAPI. Of note, signals of lipofuscin autofluorescence were detected in 488 nm, 543 nm and 633 nm but not 405 nm channels. Old EPCs were from same clone of young with additional 9 passages. Images were acquired by Leica SP8 confocal microscope. Scale bar, 50 µm.

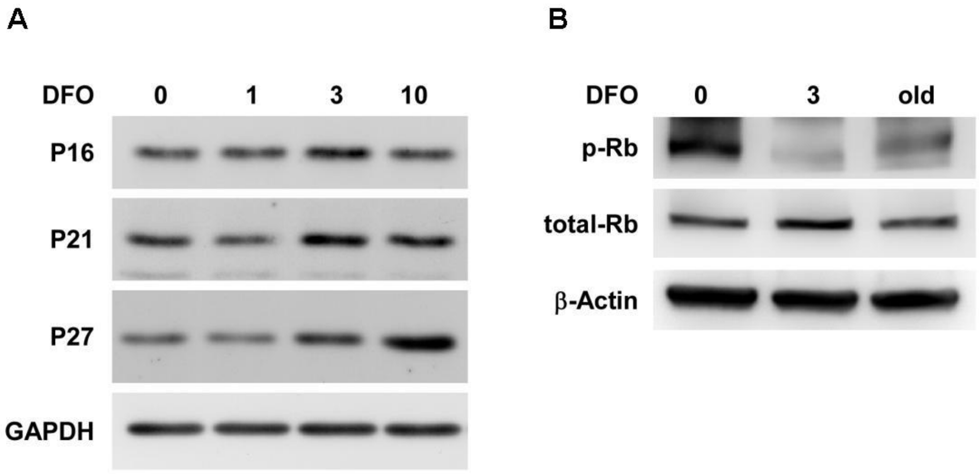

Supplementary Figure 2. Effects of DFO on the expression of senescence-related protein expression. (**A**) Young EPCs were treated with indicated concentration (μM) of DFO for 4 days. (**B**) Young EPCs were treated with 3 μM of DFO for 4 days. Lysates of old EPCs (old) from the same clone of young EPCs with additional 9 passages were loaded for comparison. Whole cell lysates were harvested and resolved by SDS-polyacrylamide gels. Western blots were probed with indicated antibodies. GADPH and β-Actin are for loading control. Same results were obtained from experiments repeated for three times.

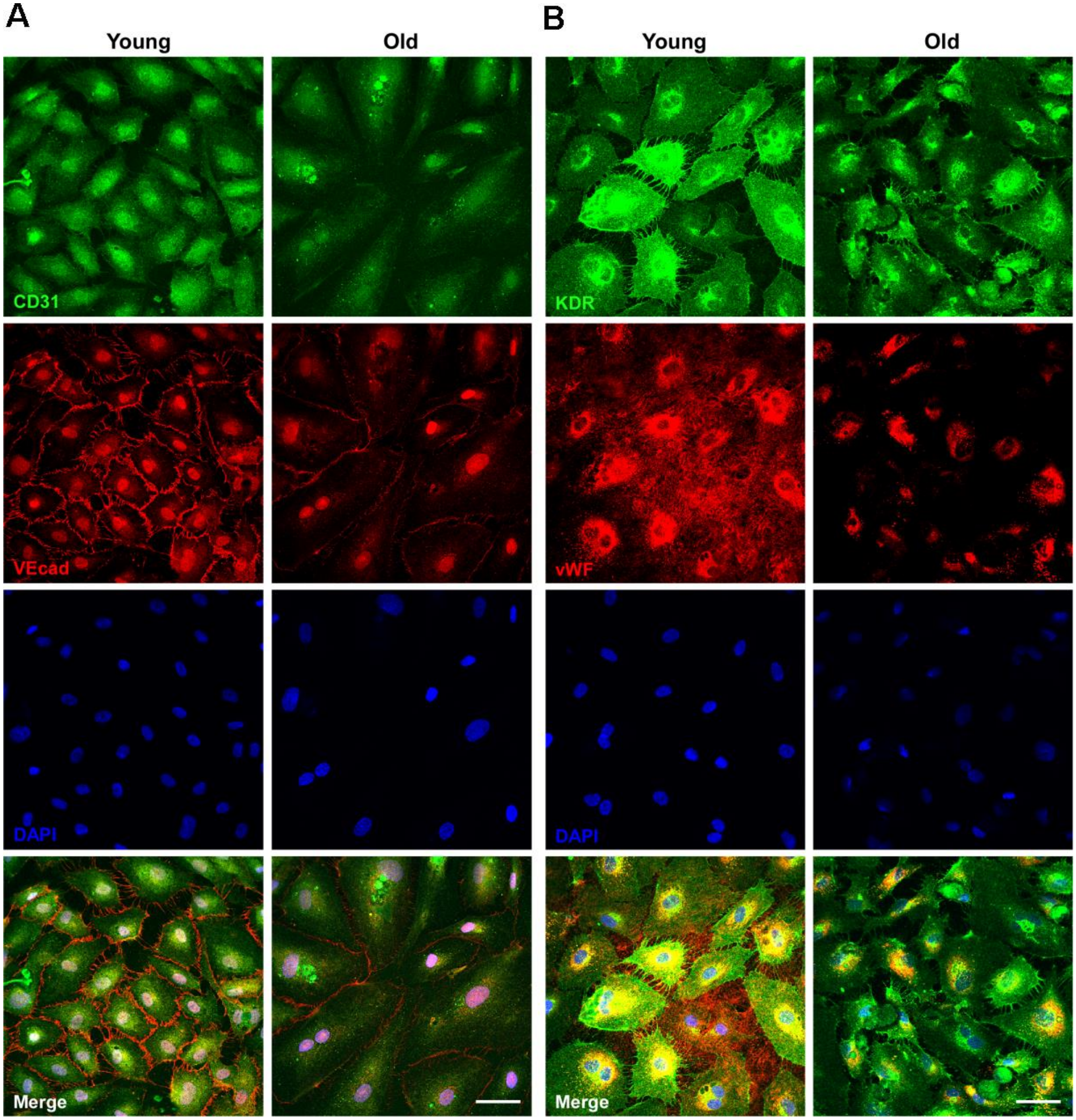
A
Young
Old
B
Young
Old
CD31
VEcad
DAPI
Merge
KDR
vWF
DAPI
Merge

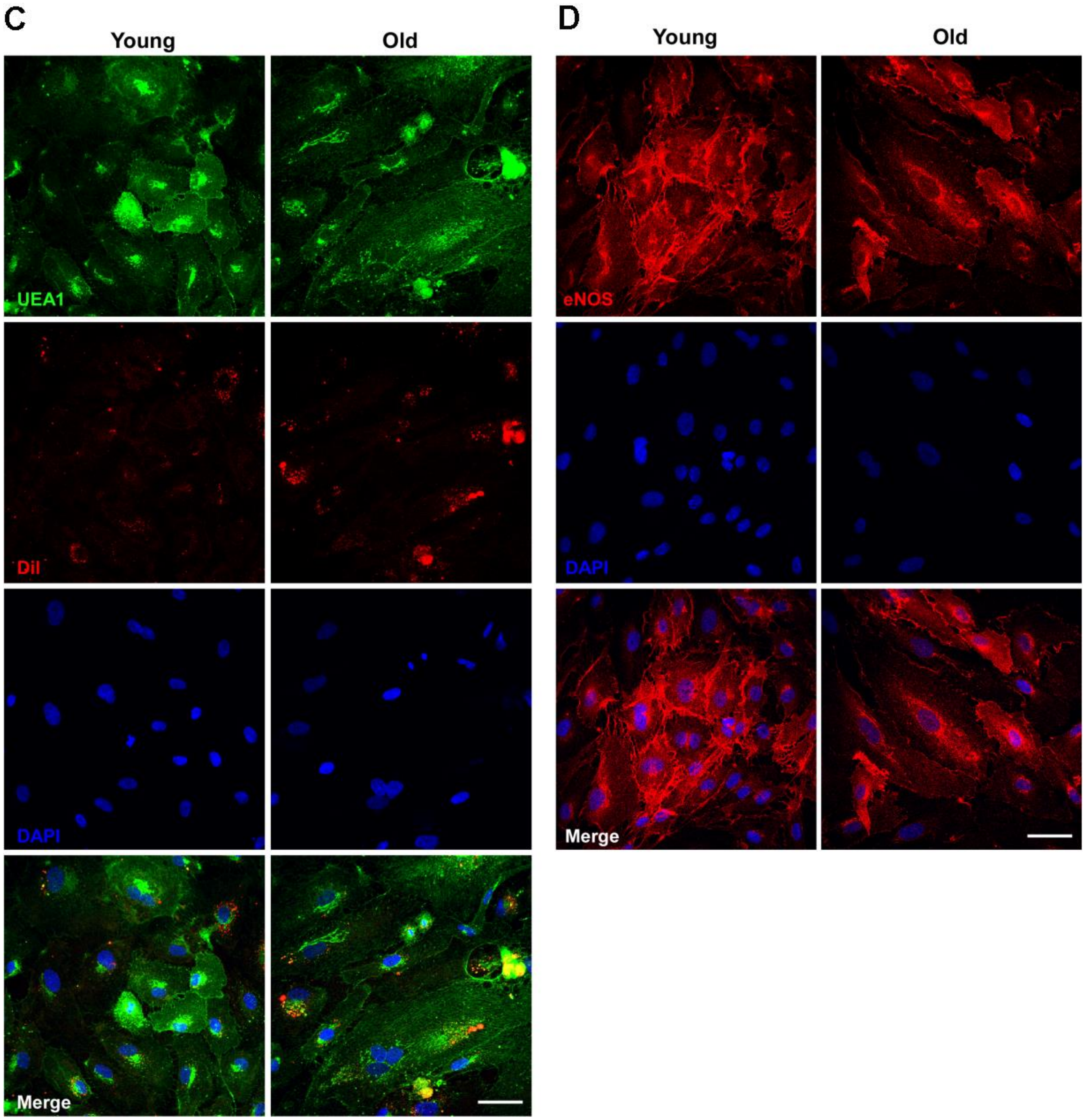

Supplementary Figure 3. Characterization of young and old EPCs. Same clone of young EPCs (P10) with additional 8 passages of old EPCs were harvested and fixed with 4% paraformaldehyde for 10 min. After 3 times of PBS-0.2% Triton X-100 washes and 10% horse serum blocking for 1 hr, cells were stained with indicated antibodies. (**A**) Cell peripherals were delineated by Ve-cdherin (VEcad) and CD31 is a EPC marker. (**B**) Cells were stained with KDR for EPCs. vWF is expressed in endothelial cells. (**C**) Uptake of DiI-acLDL and the staining of UEA1 are hallmarks of EPCs. (**D**) eNOS is a endothelial marker, correlated to angiogeneic activity. Images were acquired by Leica SP8 confocal microscope. Scale bar, 50 μm.
